# Genetic diversity and population structure of non-descript cattle in South African smallholder systems

**DOI:** 10.3389/fgene.2025.1535730

**Published:** 2025-03-18

**Authors:** M. P. Ramoroka, M. D. MacNeil, F. W. C. Neser, S. F. Lashmar, M. L. Makgahlela

**Affiliations:** ^1^ Department of Animal Science, University of the Free State, Bloemfontein, South Africa; ^2^ Agricultural Research Council, Animal Production, Irene, South Africa; ^3^ Delta G, Miles, MT, United States

**Keywords:** adaptability, crossbreeding, genetic characterization, non-descript, singlenucleotide polymorphism

## Abstract

The genetic background and characteristics of South African smallholder cattle populations remain largely unknown. These cattle exhibit remarkable adaptability to challenging environments with minimal inputs from farmers, making them a valuable genetic resource for sustainable farming. This study aimed to genetically characterize non-descript cattle kept in smallholding systems using single-nucleotide polymorphism (SNP) markers. A total of 188 non-descript smallholder beef cattle were sampled from seven South African provinces; Eastern Cape (n = 27), Free State (n = 28), Gauteng (n = 13), KwaZulu-Natal (n = 29), Limpopo (n = 34), North West (n = 44) and Northern Cape (n = 10). In addition, samples were obtained from Afrikaner (n = 42), Bonsmara (BON; n = 46), Boran (n = 20), Brahman (n = 96), Drakensberger (n = 25), Hereford (n = 31), Holstein (HOL; n = 29), Nguni (n = 59) and Shorthorn (n = 35) to serve as reference populations. Quality control of the original SNP data removed less informative animals and SNPs, which resulted in a final data set consisting of 185 animals and 119,392 SNPs. Principal coordinate analysis, ancestry, and genomic diversity statistics revealed moderate to high levels of diversity within smallholder cattle and substantial relationship with commercial beef cattle (i.e., Afrikaner, Bonsmara, Brahman, Drakensberger, Hereford, Holstein and Nguni). In North West province, there was tendency towards greater influence of Bonsmara, whereas in KwaZulu Natal the cattle were more closely related to Holstein. The smallholder populations were shown not to be unique, likely due to indiscriminate hybridization with the commercial breeds. Among the provinces, estimates of observed heterozygosity (H_O_) ranged from 0.328 ± 0.001 to 0.395 ± 0.001, while expected heterozygosity (H_E_) ranged from 0.326 ± 0.001 to 0.389 ± 0.000. Inbreeding levels were low, with (mean ± standard error) per-province inbreeding coefficients (F_IS_) ranging from −0.023 ± 0.009 to 0.133 ± 0.0254. The low F_ROH_ (<0.05) across all populations indicate a more diverse population, which is less likely to express deleterious recessive traits. Estimates of the population differentiation fixation index (F_ST_) indicated greater genetic distance between animals from KwaZulu natal and Gauteng provinces (F_ST_ = 0.083) and less distance between the animals from Eastern Cape and Free State provinces (F_ST_ = 0.010), suggesting a closer genetic relationship probably as a result of the proximity of the latter provinces and hence trans-boundary use of bulls. These findings suggest indiscriminate crossbreeding in smallholder cattle within and across the provinces of South Africa. The results provide foundational information for the transfer of technology for targeted breeding programs to smallholder farmers.

## 1 Introduction

In South Africa, smallholder farmers manage approximately 40% of the national cattle population, playing a crucial role in food security and sustaining rural livelihoods (Köhler-Rollefson, 2004). These farmers predominantly rely on resilient indigenous breeds from the Sanga group, such as the Afrikaner, Nguni, and Drakensberger, which are well-adapted to the environment with harsh conditions, including nutritional, parasitic, and pathogenic challenges (Scholtz, 2010; Nyamushamba et al., 2017). The hardiness of these breeds makes them essential to smallholder production systems, contributing to both local food security and rural economic stability (Kumar, 2019; Nyamushamba et al., 2017). Furthermore, indigenous cattle help preserve genetic diversity and may hold the potential to improve commercial breeds in the future (Mapiye et al., 2019).

Unregulated crossbreeding between indigenous and exotic breeds has been a longstanding practice, resulting in the widespread emergence of non-descript cattle, which now constitute up to 66.4% of smallholder herds (Bessa et al., 2009; Mapiye et al., 2009; Scholtz et al., 2008). This shift poses significant risks to the genetic integrity of traditional breeds and threatens to erase valuable genetic resources (Khombe, 2002; Nyamushamba et al., 2017). Despite their prevalence, the genetic diversity and adaptive traits of non-descript cattle remain poorly understood, limiting their potential for strategic breeding and conservation (Scholtz et al., 2008). Inadequate knowledge of breed composition and the relationships within and between populations could lead to the loss of local genetic resources and increased risks of inbreeding depression (Rege et al., 2007; Rewe et al., 2009).

Traditionally, non-descript cattle populations were characterized by their phenotypic traits such as body size and coat color (Teneva et al., 2009). However, recent technological advances in the field of genomics, such as SNP genotyping and whole-genome sequencing, facilitated their genetic characterization, offering a more precise means of assessing genetic diversity, demographic history, and phylogenetic relationships with other local and international breeds (Gama and Bressan, 2011; Shirasawa et al., 2012; Cendron et al., 2024). This study aims to genetically characterize the non-descript cattle populations of South African smallholder farms, providing crucial insights into their genetic history and enabling the identification of population variation and breed origin.

## 2 Materials and methods

The study was approved by the ethics committee of the Agricultural Research Council-Animal Production (Ethics number: APIC18/03).

### 2.1 Sampling and genotyping

**FIGURE 1:**
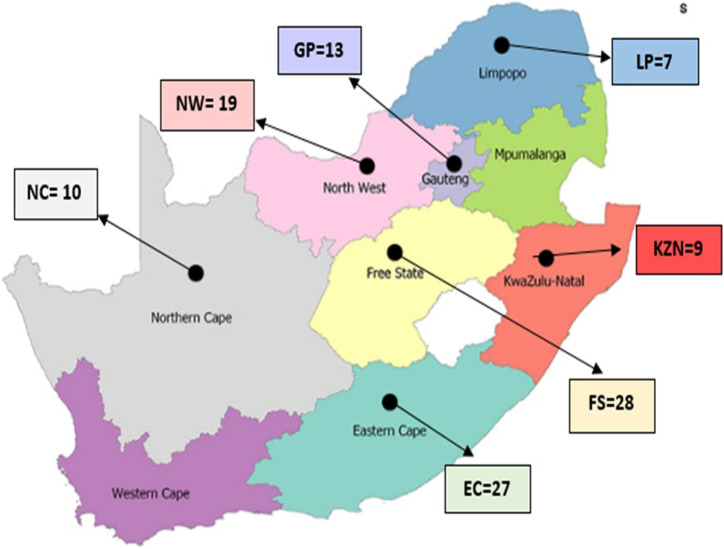
Map of South Africa indicating the provinces and geographical locations of the smallholder populations studied.

A total of 188 hair samples were collected from non-descript smallholder (SHD) cattle from rural areas in the Eastern Cape (EC; n = 27), Free State (FS; n = 28), Gauteng (GP; n = 13), KwaZulu-Natal (KZN; n = 29), Limpopo (LP; n = 34), North West (NW; n = 44) and Northern Cape (NC; n = 10) provinces. Animals were randomly selected from several SHD cattle farmers within each province. Villages were identified based on consultations with local agricultural extension officers to target areas with a strong presence of non-descript cattle populations. Multiple villages per region were included to ensure a broad representation of the non-descript cattle. Farmer participation was voluntary, and efforts were made to include a diverse range of cattle, specifically targeting older animals (≥10 years of age), to provide a representative sample of the SHD cattle population. Samples were not collected in the Western Cape (WC) and Mpumalanga (MP) due to the scarcity of non-descript cattle and logistical challenges, respectively. Figure 1 illustrates the geographical areas in SA where samples were collected (specific sampling sites are represented by black marks).

Genotyping was conducted at the ARC-Biotechnology Platform. The GeneSeek^®^ Genomic Profiler™ (GGP) bovine 150 K SNP panel (featuring over 130, 000 SNPs for *Bos taurus* x *Bos indicus*) was used to obtain the genotypes from SHD populations. Genotyped samples were imaged with the Illumina^®^ iScan Reader and a Genome Studio plug-in. The raw Illumina^®^ Genome Studio v2.0 output files were converted into PLINK v1.9 software (Purcell et al., 2007) input files for quality check and further downstream analysis. Genotypes for Afrikaner (AFR; n = 42), Bonsmara (BON; n = 46), Brahman (BRA; n = 96), Drakensberg (DRB; n = 25), Holstein (HOL; n = 29) and Nguni (NGU; n = 59) breeds were obtained from Beef Genomics Program (BGP). Additionally, Boran (BOR; n = 20), Hereford (HFD; n = 31), and Shorthorn (SHT; n = 35) were obtained from the Web-interfaced next-generation Database dedicated to genetic Diversity Exploration (WIDDE (http://widde.toulouse.inra.fr/widde/widde/main.do?module=cattle). These reference datasets had been genotyped using the Illumina^®^ Bovine SNP50 (BOR and HRD) and GeneSeek^®^ Genomic Profiler™ (GGP) bovine 150 K SNP panels (AFR, BON, BRA, DRB, HOL, and NGU). In preparation for the joint analysis, the SNPs common to both the 50K and 150 K reference datasets were extracted. Since the SHD dataset was also genotyped using the bovine 150 K SNP array, the common SNPs were similarly extracted from that dataset as well. A total of 49,564 common SNPs were identified between the 50K and 150 K datasets. The reference and SHD population datasets were merged using the--bmerge command in PLINK v1.9 software (Purcell et al., 2007).

### 2.2 Data editing

Data were edited with PLINK v1.9 (Purcell et al., 2007) to discard low-quality markers and samples. Individuals with sample call rate less than 90% were removed as were non-autosomal, unmapped, and duplicated SNPs. In addition, individual SNP were required to have call rate greater than 95%, minor allele frequency (MAF) greater than 5%, and to exhibit Hardy-Weinberg equilibrium (P > 0.0001). After editing, the final SHD data set consisted of 185 individuals and 119,392 SNPs was retained for statistical analysis.

### 2.3 Analysis of molecular variance and genetic differentiation

The minor allele frequency (MAF), observed heterozygosity (H_O_), expected heterozygosity (H_E_), and inbreeding coefficient (F_IS_) were estimated for the study populations using the PLINK v1.9 software (Purcell et al., 2007; www.cog-genomics.org/PLINK/1.9/). The R-package detectRUNS (Biscarini et al., 2018) was employed to detect the runs of homozygosity (ROH), which are indicative of elevated homozygosity due to mating of closely related individuals (Zeitler and Gilbert, 2024). Several parameters were used to define ROH: (i) a minimum ROH length of 500 kb, (ii) a homozygous overlap window proportion of 0.05, (iii) a minimum of 100 consecutive SNPs per ROH, (iv) a minimum SNP density of one SNP per 100 kb, (v) a maximum gap of 100 kb between consecutive homozygous SNPs, and (vi) a tolerance of up to two SNPs with missing genotypes and a maximum of one heterozygous SNP within an ROH, as described by Xu et al. (2019). The ROH were analyzed to estimate the molecular inbreeding coefficient based on ROH (F_ROH_) for each animal with *R* v4.2.2 (R Core Team, 2019). To detect differentiation among provinces, among individuals within provinces, and within individuals in the studied provinces, an analysis of molecular variance was conducted and pairwise genetic distances (F_ST_) were calculated using ARLEQUIN v3.5.2 software (Excoffier et al., 2005). The F_ST_ statistics were used to define the degree of non-random association of alleles within individuals and measure the genetic distance between the populations (Caballero and Toro, 2002).

### 2.4 Population structure analysis

Principal Coordinates Analysis (PCoA) was performed and a phylogenetic tree (Nei distance matrix) was generated in *R* v4.2.2 (R Core Team, 2019) to investigate the relationship among individuals in the SHD using the ‘ape’ R-package (Paradis et al., 2004; Popescu et al., 2012). The ADMIXTURE software (version 2.0; Alexander et al., 2009) was used to detect the number of ancestral populations the most appropriate number of founder populations (K) determined by cross-validation. Genesis software version 0.2.3 (Buchmann and Hazelhurst, 2012) was then utilized to visualize the ADMIXTURE results.

## 3 Results

### 3.1 Genetic diversity and differentiation

**FIGURE 2:**
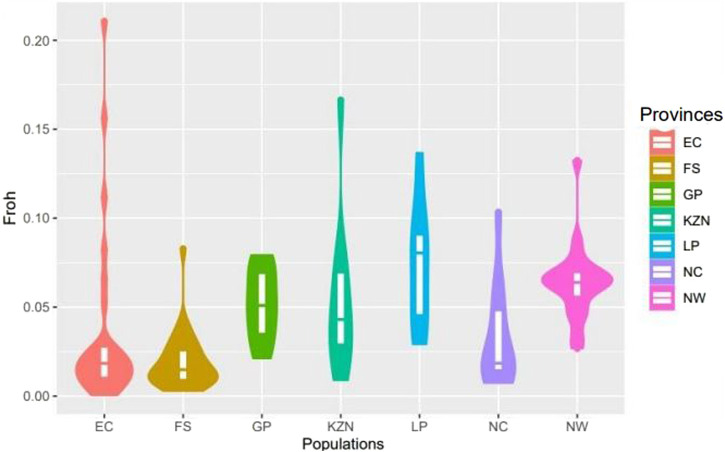
Violin plot of the inbreeding coefficients based on runs of homozygosity (FROH) in smallholder cattle populations.

The genetic diversity parameters for the SHD population are presented in Table 1. The mean and standard error of minor allele frequency (MAF) ranged from 0.247 ± 0.001 in animals from LP to 0.301 ± 0.001 in animals from the FS indicating a slightly greater presence of minor alleles in the FS and hence, a higher probability of heterozygous genotypes. This was supported by the higher level of observed heterozygosity (H_O_) in the FS (0.395 ± 0.001) compared to LP (0.328 ± 0.001). Except for animals from the EC province, all animals from each of the other provinces exhibited gains in diversity (i.e., H_O_ > H_E_), and corresponding low levels of inbreeding, with F_IS_ values ranging from −0.023 ± 0.009 in FS to 0.133 ± 0.025 in LP. The observed F_ROH_ ranged from 0.020 ± 0.004 in NW to 0.047 ± 0.006 in KZN. The F_ROH_ across provinces were further visualized in Figure 2 wherein animals from EC and KZN displayed the widest range of F_ROH_ values, with some individuals having very high inbreeding coefficients (up to 0.20), indicating substantial variation in inbreeding levels. Animals from FS, GP, LP, NC and NW showed narrow ranges of F_ROH_ values, suggesting relatively low to moderate levels of inbreeding.

**TABLE 1:** Summary of minor allele frequency (MAF), observed (H_O_) and expected (H_E_) heterozygosity, inbreeding coefficient (F_IS_) and inbreeding coefficient based on ROH (F_ROH_) for the provincial non-descript cattle.

Populations	Number of individuals	MAF ±se	H_O_ ± se	H_E_ ± se	F_IS_ ± se	F_ROH_ ± se
EC	27	0.293 ± 0.001	0.375 ± 0.001	0.382 ± 0.000	0.029 ± 0.015	0.033 ± 0.003
FS	28	0.301 ± 0.001	0.395 ± 0.001	0.389 ± 0.000	−0.023 ± 0.009	0.023 ± 0.000
GP	13	0.256 ± 0.001	0.358 ± 0.001	0.338 ± 0.001	0.073 ± 0.005	0.027 ± 0.000
KZN	29	0.258 ± 0.001	0.347 ± 0.001	0.341 ± 0.001	0.102 ± 0.022	0.047 ± 0.006
LP	34	0.247 ± 0.001	0.328 ± 0.001	0.326 ± 0.001	0.133 ± 0.025	0.0039 ± 0.004
NC	10	0.270 ± 0.001	0.368 ± 0.001	0.355 ± 0.001	0.053 ± 0.015	0.024 ± 0.002
NW	44	0.259 ± 0.001	0.355 ± 0.001	0.342 ± 0.001	0.084 ± 0.007	0.020 ± 0.004
All	185	0.271 ± 0.001	0.364 ± 0.001	0.346 ± 0.001	0.064 ± 0.014	0.025 ± 0.001

Eastern Cape (EC), Free State (FS), KwaZulu-Natal (KZN), Northern Cape (NC), Gauteng (GP), Limpopo (LP) and North West (NW).

The AMOVA revealed that 94% of the variation in genotypes was present within the individual animals (Table 2). Only 1% of the variation was explained by individuals within provinces, and the difference between provinces explained 5% of the variation. The high percentage of variation within individuals indicates their genetic diversity, which can be expected in outbreeding species where individuals are not highly related.

**TABLE 2:** The analysis of molecular variance among smallholder populations.

Source of variance	Sum of squares	Variance components	Percentage variation
Among populations	188,366	593	5
Among individuals within populations	1,366,660	114	1
Within individuals	1,427,982	12,310	94
Total	2,983,007	13,017	

Estimates of pairwise F_ST_ are presented in Table 3. The results indicated greater genetic differentiation (pairwise F_ST_ = 0.083) between animals from the non-adjacent KZN and GP provinces compared to those from the adjacent EC and FS (pairwise FST = 0.010) provinces, suggesting some potential differentiation resulting from geographic distance.

**TABLE 3:** Genetic differentiation (F_ST_) among pairs of smallholder populations.

	EC	FS	GP	KZN	LP	NC	NW
EC	**0**						
FS	*0.010	**0**					
GP	0.051	0.046	**0**				
KZN	0.019	0.034	**0.083	**0**			
LP	0.036	0.043	0.027	0.053	**0**		
NC	0.014	0.016	0.047	0.038	0.027	**0**	
NW	0.045	0.043	0.070	0.070	0.062	0.024	**0**

SHD, population: Eastern Cape (EC), Free State (FS), KwaZulu-Natal (KZN), Northern Cape (NC), Gauteng (GP), Limpopo (LP) and North West (NW).

Bold value indicates: *Lowest pairwise FST value (i.e. least differentiated); **Highest pairwise FST value (i.e. most differentiated).

**FIGURE 3:**
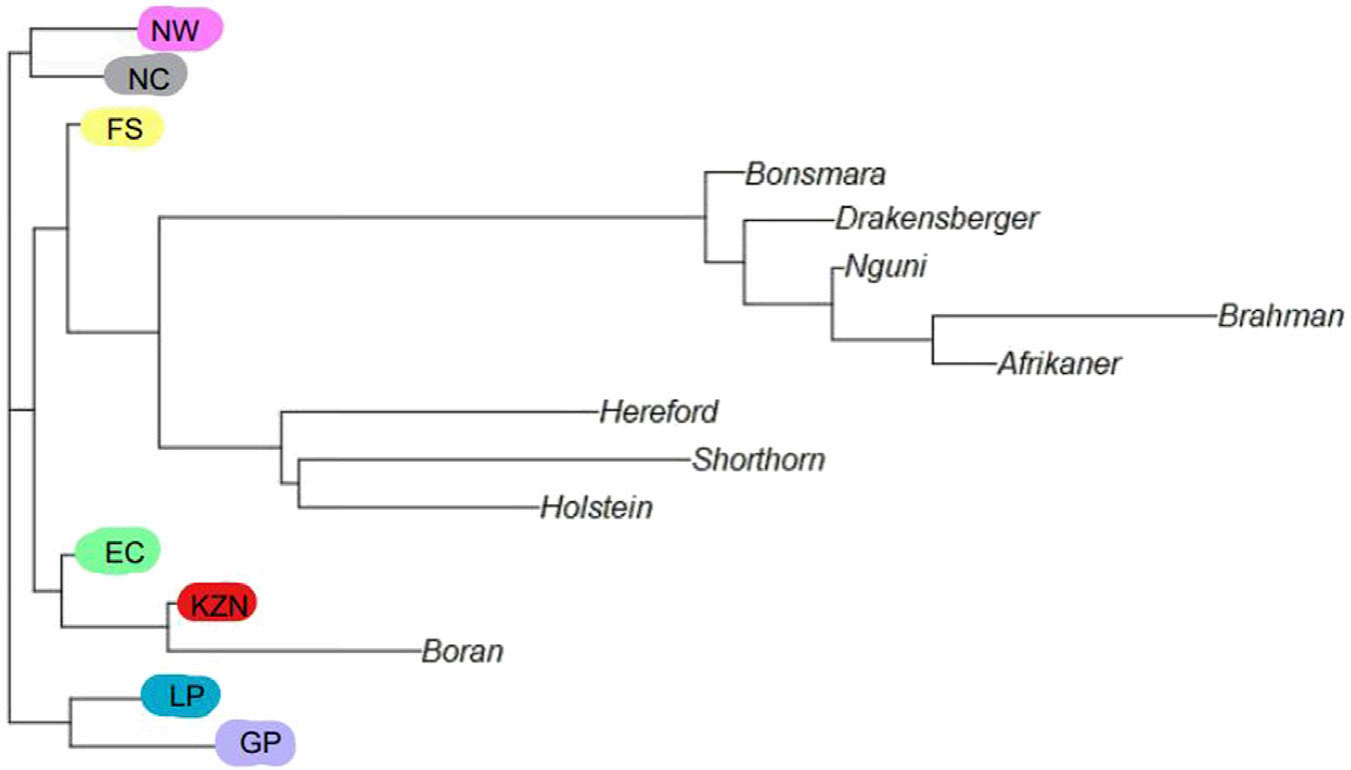
Phylogenetic tree between reference and smallholder population: Eastern Cape (EC), Free State (FS), KwaZulu-Natal (KZN), Northern Cape (NC), Gauteng (GP), Limpopo (LP) & North West (NW).

The phylogenetic tree (Figure 3) provides valuable insights into the genetic composition and relationships between the non-descript cattle and established commercial and indigenous breeds. The non-descript cattle in the FS were more closely tied to the recognized breeds indicating that those non-descript cattle were more influenced by them than in the other provinces. The NW, and NC populations are quite distinct from the commercial breeds. While, KZN and EC show a notable connection to the Boran breed. The cattle in LP and GP form a distinct branch, separated from both commercial and indigenous breeds.

### 3.2 Genomic relatedness and ancestry estimation

**FIGURE 4:**
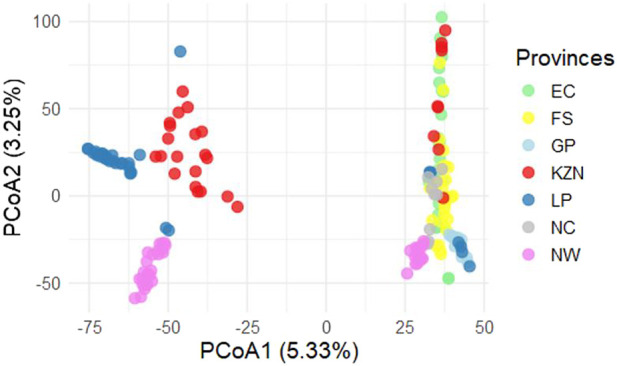
Principal coordinates analysis (PCoA) for Smallholder cattle population (SHD). Eastern Cape (EC), Free State (FS), KwaZulu-Natal (KZN), Northern Cape (NC), Gauteng (GP), Limpopo (LP) & North West (NW).

The PCoA was used to determine the genomic relationships between all of the SHD cattle sampled within each location and across all of the provinces. The only provinces that showed more defined clustering (i.e., high relatedness between cattle within the province), were GP, LP, and NW; with GP and LP forming a combined cluster (i.e., showing strong genomic linkages between cattle from these provinces) (Figure 4). The SHD cattle from the remaining provinces (EC, FS, KZN, and NC) were interspersed, indicating low relatedness within provinces, however, some strong relatedness between individual animals across provinces. The first coordinate accounts for 5.33% of the total genetic variation in the population andhas an eigenvalue of 48.23, indicating that it captures a substantial proportion of the total variance in the SHD cattle. This large eigenvalue suggests that first coordinate represents a major underlying pattern or relationship within the SHD cattle, potentially linked to a dominant trait or combination of traits influencing the overall population structure. The second coordinate, with an eigenvalue of 22.53, explains notably less variance than the first. However, this substantial eigenvalue suggests that the second coordinate may capture a distinct biological or environmental factor contributing to population diversity, potentially reflecting regional or adaptive traits. The third through sixth coordinates, with eigenvalues of 10.85, 10.18, 7.65 and 7.25, respectively, reflect further elucidated structural details. These components likely represent additional, more subtle sources of variation within the SHD cattle, potentially associated with traits or specific environmental interactions. Together, these components provide a better view of the underlying genetic diversity across the provinces of South Africa with those that are more divergent along the first coordinate being less genetically related. In this case, cattle from LP and NW have distinct genetic differences compared to the others. The second coordinate represents 3.25% of the genetic variation. The EC, KZN, FS, and NC populations appear to group relatively close to each other indicating that they are genetically similar. This close clustering is interpreted to suggest these populations share a common genetic background.

**FIGURE 5:**
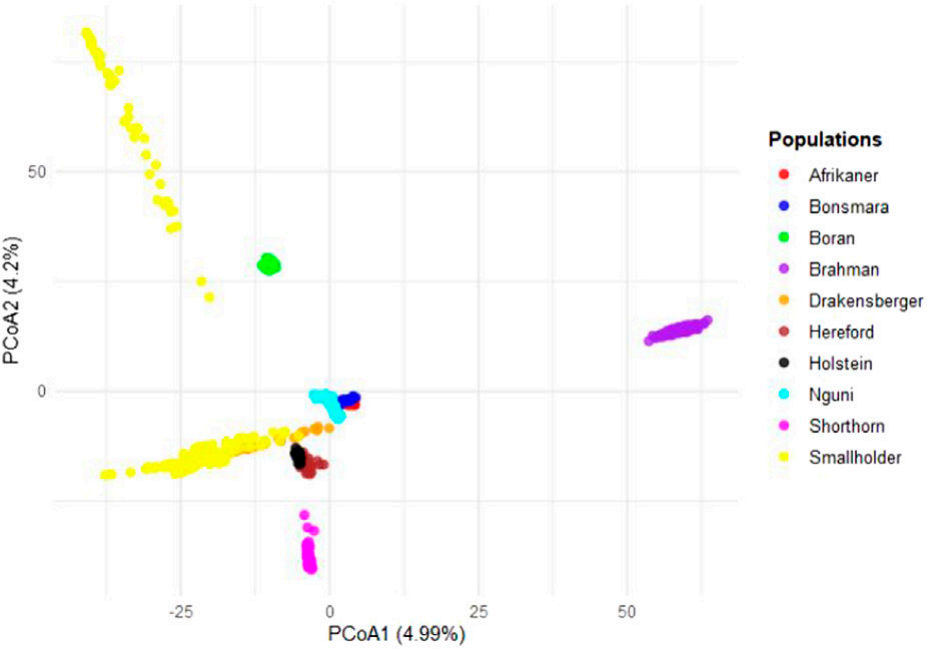
Principal coordinates analysis (PCoA) for smallholder and commercial cattle breeds.

Secondarily, the SHD cattle were combined into a single group and compared to recognized commercial breeds (Figure 5) to identify possible genomic relationships between non-descript cattle and the well-recognized breeds of cattle. Results showed strong genomic relatedness to the larger Sanga (*B. taurus africanus*) cluster, indicating a predominance of Sanga genetics within the non-descript cattle sampled.

**FIGURE 6:**
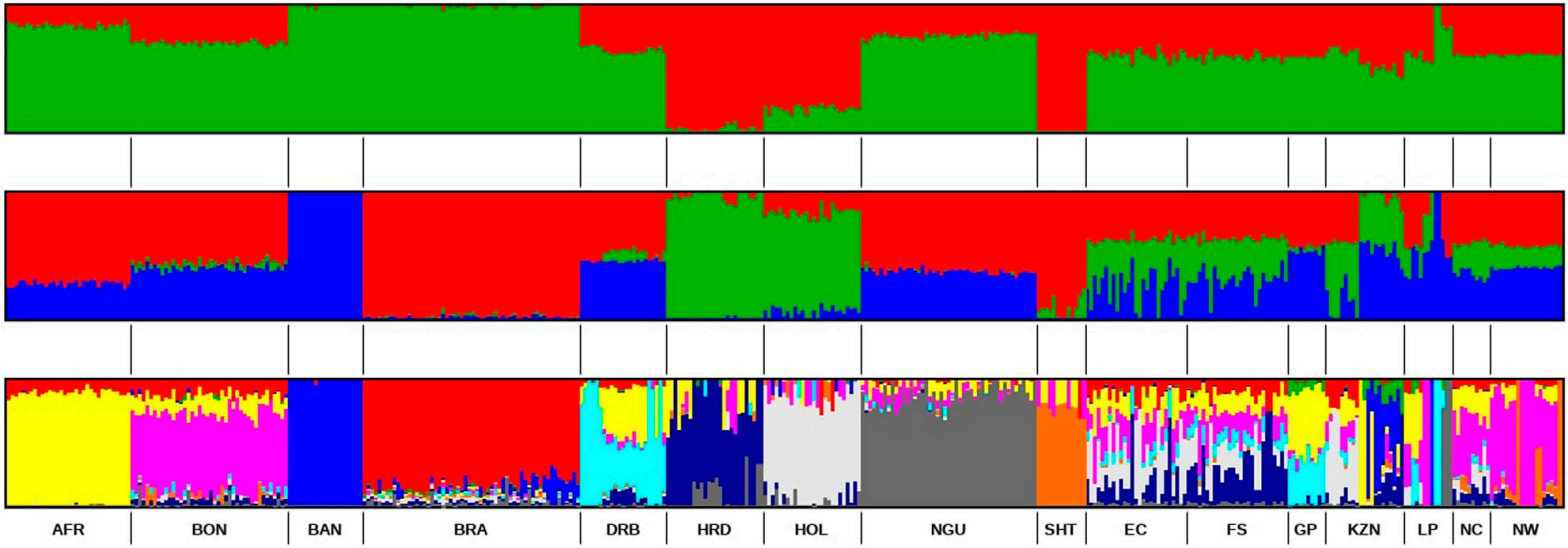
ADMIXTURE-based clustering K = 2, K = 3 and K = 10. Reference population: Afrikaner (AFR), Bonsmara (BON), Boran (BAN), Brahman (BRA), Drakensberg (DRB), Hereford (HFD), Holstein (HOL), Nguni (NGU) & Shorthorn (SHT). Smallholder population: Eastern Cape (EC), Free State (FS), KwaZulu-Natal (KZN), Northern Cape (NC), Gauteng (GP), Limpopo (LP) & North West (NW).

The ancestral composition of the studied SHD population was further investigated using ADMIXTURE software (Alexander et al., 2009), which aimed to detect the maximum possible number of ancestral populations that contribute to the observed ADMIXTURE within individuals (Khanyile et al., 2015). Based on the population structure analysis (Figure 6) at K = 2, the SHD populations exhibited shared European taurine and Sanga breeds ancestry. At K = 3, the results revealed an additional ancestral contribution from *B. indicus*.

The most probable number of distinct ancestral populations was determined to be 10. The analysis revealed that the SHD cattle from EC, FS, NC, and KZN shared ancestry with Afrikaner, Bonsmara, Brahman, Drakensberg, Hereford, Holstein, and Nguni populations. Whereas, cattle from GP and LP were shown to have similar genetic backgrounds to Afrikaner, Bonsmara, and Drakensberg. Finally, the cattle held by smallholder farmers in NW were found to have Afrikaner, Bosmara, Brahman, Hereford, and Shorthorn ancestry.

Bonsmara is the most prevalent commercial beef breed in South Africa. Thus, it is not surprising that Bonsmara was at least moderately represented in the cattle of smallholder farmers across all of the provinces. Its greatest representation was in the NW cattle population wherein it exhibited 67% shared ancestry followed by the LP and NC populations with 39% and 40% shared ancestry, respectively (Table 4). While the cattle held by smallholder farmers in GP shared 30% ancestry with the Drakensberger and 42% with the Afrikaner. In EC and KZN the cattle of smallholders showed 31% and 17% shared ancestry with the Holstein, respectively. In FS the cattle held by smallholder farmers exhibited 30% ancestry with Hereford, along with 15% contributions from both the Afrikaner and Bonsmara breeds.

**TABLE 4:** Shared ancestry (%) between the SHD cattle populations and existing beef breeds.

	AFR	BON	BAN	BRA	DRB	HRD	HOL	NGU	SHT
EC	15	18	1	11	6	16	31	1	1
FS	16	17	0	10	9	30	16	1	1
GP	42	24	0	2	30	1	0	0	0
KZN	16	19	22	7	1	12	17	0	7
LP	7	48	4	10	13	0	6	12	0
NC	23	40	0	6	3	10	12	1	3
NW	7	67	0	3	1	4	1	0	19

Reference population: Afrikaner (AFR), Bonsmara (BON), Boran (BAN), Brahman (BRA), Drakensberg (DRB), Hereford (HFD), Holstein (HOL), Nguni (NGU) and Shorthorn (SHT). Smallholder, population: Eastern Cape (EC), Free State (FS), KwaZulu-Natal (KZN), Northern Cape (NC), Gauteng (GP), Limpopo (LP) and North West (NW).

## 4 Discussion

In this study, we performed a genomic characterization of non-descript cattle in South African smallholder production systems investigating their genomic composition, level of genetic diversity, and the relatedness within and between the populations in different provinces of South Africa. The purported ability of indigenous breeds to adapt to a changing environment may be a result of their genetic diversity, which further assists with improvement and conservation efforts (Chokoe et al., 2020). Information on both internal (variation in genetic makeup within a single population) and external (variation in genetic makeup between different populations) genetic diversity and population structure becomes crucial when designing genetic improvement and conservation strategies at the national and international levels (Tittensor et al., 2014). To maximize the productivity of smallholder systems, Ojango et al. (2014) argued that the use of genomic data to assess genetic diversity and population structure could produce optimal recommendations considered by farmers. Additionally, it provides smallholder industries in developing regions an opportunity to participate in genetic enhancement programs (Maake, 2020).

According to Hlophe (2011), genetic variation within populations is crucial to allow individuals to adapt to changing environments. Furthermore, Thornton (2010) suggested that the observed genetic variation presents an opportunity to implement genetic improvement programs for smallholder farmers. The SHD cattle had MAF averaging 0.27 across all provinces. Makina et al. (2015) observed MAF for overall chromosomes was 0.25 for Afrikaner, 0.26 for Nguni, 0.27 for Drakensberger and 0.26 for Bonsmara; results which are similar to those found for the smallholder cattle in this study. According to Botero et al. (2014), the potential for the animal to adapt to environmental changes in the future will decline as the level of heterozygosity decreases and alleles are lost. The cattle sampled in this study have demonstrated the potential for long-term survival (over 10 years) and adaptation to harsh environmental conditions. The genetic diversity within the cattle held by smallholder farmers was measured based on observed (H_O_) and expected (H_E_) heterozygosity and the inbreeding coefficient (F_IS_). The average mean H_E_ observed in the current study was lower than reported for stud animals of the SA Drakensberger (H_E_ = 0.36) and the composite Bonsmara (H_E_ = 0.37; Van Marle-Köster et al., 2021), as well as for non-descript dairy cattle in South Africa (H_E_- = 0.4; Maake, 2020). However, it was higher than the H_E_ of 0.19 observed for non-descript cattle in Bangladeshi (Bhuiyan et al., 2021) and the 0.34 reported for Nguni cattle (Van Marle-Köster et al., 2021). Since the observed heterozygosity (Ho) is slightly greater than the expected heterozygosity (H_E_) in SHD populations (H_O_ > H_E_), this suggests a gain in diversity. Thus, the cattle held by smallholder farmers have a relatively high level of genetic diversity, which could indicate a more diverse gene pool or a different breeding history which can be important for long-term adaptability and resilience. However, the difference between H_O_ and H_E_ is very small, indicating only a minor gain in diversity, and the populations are likely close to Hardy-Weinberg equilibrium. According to Sharma et al. (2016), the difference between H_O_ and H_E_ is due to non-random mating among the individuals of the population. Higher heterozygosity contributes to greater genetic diversity, which enhances the capacity of cattle to adapt to fluctuating environmental conditions (Kanaka et al., 2023). The increased heterozygosity observed in the smallholder cattle provides a genetic basis for the advantages of heterosis. Heterosis refers to the phenomenon where crossbred individuals exhibit superior performance, particularly in traits like growth rate, fertility, and adaptability (Falconer and Mackay, 1996). This effect is important in low-input, smallholder systems where environmental stressors such as limited feed, extreme climates, and disease pressures are prevalent. The study’s results showed low inbreeding levels (F_IS_ and F_ROH_), supporting the hypothesis that extensive crossbreeding reduces the risk of inbreeding, as suggested by Radhika et al. (2018). The inbreeding coefficient derived from ROH (F_ROH_) is, on average, 0.025 ± 0.001 across all of the cattle from smallholder farmers. Since F_ROH_ represents the probability of regions being identical by descent (Kardos et al., 2018), the combination of low F_IS_ and F_ROH_ values suggests that SHD populations have large effective population sizes. However, the cattle from EC and KZN include several outliers with higher F_ROH_ values, indicating that certain individuals are substantially inbred. The variability in F_ROH_ highlights opportunities to exploit heterosis by identifying and crossing genetically diverse individuals. In contrast, farmers whose cattle have less range in F_ROH_, such as those in the FS, GP, LP, NW and NC, may benefit from introducing new genetic material to mitigate the risk of future genetic bottlenecks and maintain genetic diversity. Moreover, heterosis can mitigate the deleterious effects of inbreeding, a common concern in isolated or small populations (Söderquist et al., 2020). The introduction of genes from diverse breeds, including Sanga and taurine cattle, creates a buffer against the negative effects of inbreeding, contributing to the overall genetic health and adaptability.

The SHD cattle had a moderate level of genetic diversity across the provinces of South Africa, which may be an indication of extensive crossbreeding. According to Ojango et al. (2014), the admixture of diverse populations and natural selection for adaptation is supported by high levels of genetic diversity. Smallholder animals are genetically diverse and can adapt to various production systems, and the value of diversity in this sector has been strongly emphasized (Morrison, 2007; Mdladla, 2016). Understanding the genetic diversity of the SHD cattle population is crucial because it helps identify the range of genetic traits within the population. This diversity is important for achieving maximum productivity, as it allows for the selection of cattle with desirable traits such as disease resistance, growth rate, and adaptability to local environments. It ensures that breeding programs maintain a healthy and sustainable population, prevent inbreeding, and support the long-term viability of cattle populations in smallholding systems. The evaluation of genetic diversity ensures that specific breeds are attained for rural development initiatives and genetic improvement programs (Chagunda et al., 2018).

The genetic variation is distributed within and between SHD cattle populations. The highest percentage variance (94%) of the SHD population was contributed by (and could be explained by) the genetic diversity within individuals (i.e., the genome-level diversity per animal) with only small percentages (5% and 1%, respectively) accounting for among-population (i.e., across provinces) and among-individuals within populations (i.e., within provinces). This sub-division of the explained variation was in concordance with observations by Groeneveld et al. (2010). The variation among SHD populations could be attributed to their geographical area, farmer’s preferences, socio-cultural aspects, natural processes of mutation, and adaptation to the different ecological zones of SA. The variation within province specific SHD populations is supported by the population structure analysis and indicates a well-diverse population with a low risk of inbreeding. Although positive inbreeding was observed in SHD cattle populations (EC, GP, KZN, LP, NC, and NW), the AMOVA results show that the population can be improved through breeding selection.

In this study, the genetic relatedness (PCoA), ancestry (ADMIXTURE), and genetic diversity estimators concordantly supported the genetic diversity in and amongst South African SHD cattle populations. According to Pritchard et al. (2000), population structure examines the variation within a population by considering factors such as individual differences, migration patterns, and the occurrence of crossbreeding. The population structure of individuals from EC, FS, KZN, and NC explains that most of the animals are raised in communal areas with no formalized breeding programs, where individuals mate naturally and randomly (Scholtz et al., 2008). The majority of smallholder farmers prefer to breed with bulls from indigenous breeds due to their low maintenance requirements and ability to adapt to local conditions, survive, and reproduce (Nyamushamba et al., 2017). Most cattle in the rural areas (smallholder farmers) are non-descript and crossbreds of local indigenous breeds in SA (Palmer and Ainslie, 2006). The SHD cattle populations in this study showed shared ancestry with predominantly Sanga breeds (*B. taurus africanus*) (Afrikaner, Bonsmara, Drakensberg, and Nguni) but also taurine (Hereford, and Holstein) and indicine (Brahman) breeds. The findings of this study align with those reported by Chagunda et al. (2018), who observed similar patterns in different indicine breeds among crossbred cattle in smallholder herds in Rwanda and Tanzania (Mujibi et al., 2019), as well as in India (Ahmed et al., 2019). In the North West (NW) province, Bonsmara cattle are the most readily available breed for smallholder (SHD) and emerging cattle farmers, who primarily use them in unstructured crossbreeding practices. The breed’s popularity is driven by its ability to produce superior replacement heifers, regardless of whether they are bred with a Bonsmara bull or another breed. This availability and adaptability make Bonsmara a preferred choice, contributing to its widespread use and success in smallholder farming systems. Since the establishment of the breed society in 1964, the Bonsmara has become the most popular cattle breed in South Africa (Steyl, 2024), demonstrating significant growth over the past 55 years. The appearance of the Holstein breed in the smallholder dairy (SHD) cattle populations of KZN, EC, and FS is attributable to the regional distribution of major milk producers in SA. These producers are categorized into four regions based on the prevailing production systems and the markets they serve, namely, KZN, WC, FS, and EC (Gertenbach, 2006). While, indigenous SA cattle breeds, such as the Nguni and Afrikaner, exhibit low milk productivity. Consequently, many SHD farmers prefer to crossbreed these indigenous breeds with specialized dairy breeds like the Holstein (Marshall et al., 2019). Additionally, cattle farmers have garnered interest from indigenous breeds due to their capacity to produce and reproduce under challenging environmental conditions and their natural immunity against ticks (Mapholi et al., 2014). This genetic admixture facilitates heterosis, which can lead to improved fitness traits such as disease resistance, heat tolerance, and reproductive efficiency. Similarly, Morrison. (2007) emphasized the value of heterosis in enhancing the adaptability of cattle in smallholder systems, where natural selection pressures favor resilient animals.

The current study provided evidence of a great variety of genomic contributions from ten inferred ancestral populations (K = 10) to the genetic composition of the SHD populations, and shared ancestry with recognized commercial breeds. The results indicated low genetic differentiation between SHD cattle populations with high levels of admixed within individuals, providing evidence of gene flow and shared genomic heritage with commercial beef cattle breeds (mainly indigenous Sanga breeds). The sampled SHD cattle showed a smallholder-farmer preference for Afrikaner, Bonsmara, Brahman, Hereford, Holstein, Drakensberger, and Nguni breeds. Although the sample size in this study was limited, and cannot comprehensively represent the cattle genomic make-up of the entire provinces, population genetics estimators indicated moderate to high levels of genetic diversity, and low inbreeding incidence, for cattle in this production system. The pairwise F_ST_ matrix indicated the smallest differentiation and, hence, a close genetic linkage between the EC and FS populations compared to other pairwise provincial comparisons. This observation indicates that cattle farmers from these two provinces could be sourcing cattle from the same place, as these provinces (regions) are neighbors, and transportation costs can be kept at a minimum (Mamogobo et al., 2020). On the contrary, the considerable genetic divergence between KZN and GP SHD populations suggests negligible gene flow that may be attributed to the physically distant between them. Geographic isolation may result in genetic divergence over time as each population adjusts to its own micro-environment. Furthermore, the coastal KZN is known to be dairy-focused (with the Holstein being the largest dairy breeds utilized). The study confirms that the genomic composition of the SA SHD cattle population is characterized by ADMIXTURE, with varying contributions from a gene pool of recognized breeds, facilitated by crossbreeding. Furthermore, the breed preference for crossbreeding is province or region-specific but across provinces, there is a tendency toward the predominant utilization of indigenous breeds, especially the Afrikaner, Bonsmara, Drakensberger, and Nguni, as well as the Brahman.

## 5 Conclusion

Genetic diversity and demographic structure of non-descript cattle that were distributed across seven provinces of SA in the non-commercial smallholder production systems were elucidated. This study shows that genomics plays a role in “breed-type” assignment by providing a detailed understanding of the genetic composition of cattle populations. By analyzing genomic data, it becomes possible to accurately assign cattle to specific breeds or ecotypes based on their genetic markers. In almost every province the non-descript cattle defy clear categorization, with no single breed dominating their genetic composition. Instead, these cattle are highly heterogeneous, reflecting unstructured crossbreeding among indigenous and commercial breeds. The high level of heterozygosity and low level of inbreeding observed suggests an influence of heterosis, which may contribute to the adaptability and resilience of these cattle in diverse and challenging environments. The lack of a predominant breed across provinces indicates much of the genetic variation exists among individual animals. These findings emphasize the potential of using the existing genetic diversity through informed breeding programs, focusing on enhancing key adaptive traits such as growth, disease resistance, and environmental resilience. Leveraging heterosis and genetic diversity could further optimize productivity while minimizing the risks of inbreeding. By implementing programs designed to regulate selective crossbreeding practices in smallholder systems, this issue can be mitigated, ensuring both improved cattle performance and the conservation of valuable genetic resources for future generations. By implementing programs designed to regulate selective crossbreeding practices in smallholder systems, this issue can be mitigated, ensuring both improved cattle performance and the conservation of valuable genetic resources for future generations. Introduction of community based breeding programs can help farmer to grouping and managing cattle according to their genomic profiles to enhance breeding strategies by enabling more targeted selection for desirable traits. This approach can lead to improved productivity, disease resistance, and adaptability to local conditions, ultimately maximizing the efficiency of resource use in SHD systems.

## Data Availability

The raw data supporting the conclusions of this article will be made available by the authors, without undue reservation.
