# Supplementary figures and images for: Genetic diversity and population structure of non-descript cattle in South African smallholder systems
Source: Front Genet. 2025 Mar 18;16:1535730. doi: 10.3389/fgene.2025.1535730 (PMC11960657; doi:10.3389/fgene.2025.1535730)

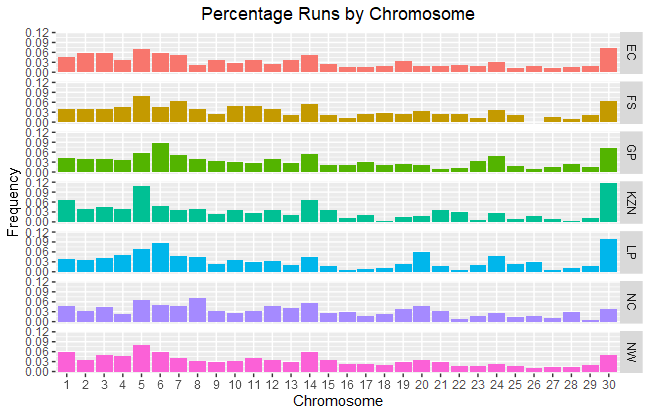

Supplement: Supplementary file 1 [file Image1.png]
